# Neurotechnologies for Human Cognitive Augmentation: Current State of the Art and Future Prospects

**DOI:** 10.3389/fnhum.2019.00013

**Published:** 2019-01-31

**Authors:** Caterina Cinel, Davide Valeriani, Riccardo Poli

**Affiliations:** ^1^Brain Computer Interfaces and Neural Engineering Laboratory, School of Computer Science and Electronic Engineering, University of Essex, Colchester, United Kingdom; ^2^Department of Otolaryngology, Massachusetts Eye and Ear, Harvard Medical School, Boston, MA, United States

**Keywords:** neuroscience, cognitive augmentation, brain-computer interfaces, decision-making, neuroergonomics

## Abstract

Recent advances in neuroscience have paved the way to innovative applications that cognitively augment and enhance humans in a variety of contexts. This paper aims at providing a snapshot of the current state of the art and a motivated forecast of the most likely developments in the next two decades. Firstly, we survey the main neuroscience technologies for both observing and influencing brain activity, which are necessary ingredients for human cognitive augmentation. We also compare and contrast such technologies, as their individual characteristics (e.g., spatio-temporal resolution, invasiveness, portability, energy requirements, and cost) influence their current and future role in human cognitive augmentation. Secondly, we chart the state of the art on neurotechnologies for human cognitive augmentation, keeping an eye both on the applications that already exist and those that are emerging or are likely to emerge in the next two decades. Particularly, we consider applications in the areas of communication, cognitive enhancement, memory, attention monitoring/enhancement, situation awareness and complex problem solving, and we look at what fraction of the population might benefit from such technologies and at the demands they impose in terms of user training. Thirdly, we briefly review the ethical issues associated with current neuroscience technologies. These are important because they may differentially influence both present and future research on (and adoption of) neurotechnologies for human cognitive augmentation: an inferior technology with no significant ethical issues may thrive while a superior technology causing widespread ethical concerns may end up being outlawed. Finally, based on the lessons learned in our analysis, using past trends and considering other related forecasts, we attempt to forecast the most likely future developments of neuroscience technology for human cognitive augmentation and provide informed recommendations for promising future research and exploitation avenues.

## 1. Introduction

*Human enhancement* refers to a very broad range of techniques and approaches aimed at augmenting body or cognitive functions, through performance-enhancing drugs, prosthetics, medical implants, human-computer teaming, etc., that result in improved characteristics and capabilities, sometimes beyond the existing human range (Moore, 2008).

For two decades many alternative definitions of human enhancement have been proposed and discussed (Parens, 1998; Bostrom, 2005; Agar, 2008; Bostrom and Roache, 2008; Moore, 2008; Savulescu and Bostrom, 2009; Cabrera, 2017), a particular bone of contention being the question of whether an intervention that simply attempts to restore function lost due to illness, injury, or disability could still be identified as enhancement.

In this paper, we will focus on a subset of means for human augmentation—*neuroscience technologies*—and only on one particular area—*human cognitive enhancement*. Our aim here is providing a snapshot of the current state of the art of neuroscience technologies for human cognitive enhancement and a motivated forecast of their most likely developments in the next two decades. Here, by *cognitive enhancement* we mean the improvement of the processes of acquiring/generating knowledge and understanding the world around us. Such processes encompass attention, the formation of knowledge, memory, judgement and evaluation, reasoning and computation, problem solving and decision making, as well as the comprehension and production of language. For these reasons, unlike previous efforts, here we choose to review applications of these technologies by the cognitive function they augment (more on this below). Readers interested in more details on recent techniques in brain function augmentation and futuristic applications are encouraged consult the comprehensive three-volume, 148-article special issue/research topic edited by Lebedev et al. (2018).

The rest of the paper is organized as follows. In section 2, we survey the main neuroscience technologies for both observing and influencing brain activity, which are necessary ingredients for human cognitive augmentation. We also compare and contrast such technologies, as their individual characteristics (e.g., spatio-temporal resolution, invasiveness, portability, energy requirements, and cost) influence their current and future role in human cognitive augmentation.

Section 3 charts the state of the art on neurotechnologies for human cognitive augmentation, keeping an eye both on the applications that already exist and those that are emerging or are likely to emerge in the next two decades. Particularly, we consider human enhancement applications in the areas of communication, cognitive enhancement, memory, decision making, attention monitoring/enhancement, situation awareness, social interactions, and complex problem solving. We cover some of the cognitive augmentation technology (language in particular) aimed at restoring lost functions in severely disable individuals, as those technologies may one day develop to the point of augmenting able-bodied and able-minded people. We also look at what fraction of the population might benefit from such technologies and at the demands they impose in terms of user training.

Because technology always develops hand in hand with society, in section 4 we briefly review the ethical issues associated with current neuroscience technologies for human cognitive augmentation. These are important because they may differentially influence both present and future research on (and adoption of) neurotechnologies for human cognitive augmentation: an inferior technology with no significant ethical issues may thrive while a superior technology causing widespread ethical concerns may end up being outlawed.

Based on the lessons learnt in our analysis and using past trends as predictors of future ones, in section 5 we attempt to forecast the most likely future developments of neuroscience technology and provide informed recommendations for promising future research and exploitation avenues.

## 2. Neuroscience Technologies for Recording and Influencing Brain Activity

The development of techniques for recording and stimulating neural activity has produced a revolution in the ability to understand the cognitive mechanisms related to perception, memory, attention, and the planning and execution of actions. However, whether or not these techniques can realistically be used for cognitive augmentation depends not only on how effective they are at detecting interpretable neural activity and/or stimulating specific target areas of the brain, but also on a number of other relevant factors. Among these is the degree of invasiveness—i.e., to what extent a technology requires introduction of instruments into the body—as well as other practical factors, including how portable or expensive technologies are, which influence their usability in everyday life for human cognitive augmentation.

In the following sections we will review these technologies with their pros and cons. For space limitations, we will not discuss in details the principles of these technologies. However, for each technique we will indicate to what degree it has helped in relation to human cognitive augmentation, leaving a more extensive description of the actual applications to section 3.

### 2.1. Technologies for Recording Brain Activity

#### 2.1.1. Non-invasive Recording Technologies

The most popular non-invasive technologies for recording neural activity are electroencephalography (EEG), functional near-infrared spectroscopy (fNIRS), functional magnetic resonance imaging (fMRI), and magnetoencephalography (MEG).

EEG records electrical activity from electrodes placed on the scalp. One of the main advantages of EEG (Niedermeyer and da Silva, 2005; Luck, 2014) is that it has very good temporal resolution, is relatively inexpensive (compared to other non-invasive recording technologies) and is portable and practical to use, an aspect that is very important when considering the usability outside the lab for cognitive augmentation. However, spatial resolution is generally low.

fMRI measures brain activity by detecting changes in the blood flow (hemodynamic response) in the brain (Logothetis et al., 2001; Buxton, 2009). It has much better spatial resolution than EEG, but temporal resolution is low. Unfortunately, fMRI needs big and expensive equipment for signal acquisition. For these reasons, despite few attempts to use it for communication (Weiskopf et al., 2004; van der Heiden et al., 2014), it is generally unsuitable for human augmentation applications (van Erp et al., 2012).

fNIRS, like fMRI, uses hemodynamic responses to assess location and intensity of brain activity (Ferrari and Quaresima, 2012). Its main advantages are that it is portable (Sagara et al., 2009; McKendrick et al., 2015), much cheaper than fMRI, and less susceptible to electrical noise than EEG. These have made this technology suitable for human cognitive augmentation applications (Coyle et al., 2007; Ayaz et al., 2013; McKendrick et al., 2014; Naseer and Hong, 2015), especially when paired with brain stimulation technologies, for example, to enhance spatial working memory (McKendrick et al., 2015). However, fNIRS has a low spatial and temporal resolutions.

Another non-invasive technology is MEG (Hämäläinen et al., 1993; Supek and Aine, 2014), which is typically used to determine the function of various parts of the brain, localize regions affected by pathology, and other medical applications. However, similarly to fMRI, MEG is bulky, requires a magnetically-shielded lab, and is expensive. For these reasons MEG is impractical for human augmentation, although some applications based on it have been proposed (Mellinger et al., 2007; van Erp et al., 2012; Ahn et al., 2013).

#### 2.1.2. Invasive Recording Technologies

Invasive technologies use electrodes directly inserted in the brain or placed on its surface. For this reason they typically allow to obtain recordings less affected by the noise and distortions induced by the scalp and skull, and with good temporal and spatial resolution. However, implanting electrodes requires brain surgery, making these techniques expensive, and presenting potential ethical issues (see section 4). One of such invasive technologies is electrocorticography (ECoG) (Wyler, 1987), a technology similar to EEG in that it measures the electrical activity generated by the neurons by means of electrodes, except that—unlike EEG—electrodes are placed directly on the cortex. Moreover, typically ECoG only measures the neural activity from a very small portion of the cortex. Nonetheless, human cognitive augmentation applications based on ECoG exist (Brunner et al., 2011; Krusienski and Shih, 2011).

Other invasive recording technologies include arrays of needle-shaped microelectrodes in the brain (Maynard et al., 1997; Oka et al., 1999). These produce good signals, only marginally affected by noise and very detailed (i.e., each electrode measures the electrical activity of one or very few neurons). Examples of invasive electrodes include ceramic-based microelectrodes developed by Gerhardt and collaborators (Hampson et al., 2003). The electrodes, thanks to their elongated structure and the presence of multiple pads on their surface, allow high-precision and high-density multi-recordings in deep brain structures (Hampson et al., 2003; Opris et al., 2015), as well as electrical stimulation (Berger et al., 2011; Hampson et al., 2013, 2018). A limitation of invasive recording tools is that they typically cover only very limited regions of the brain, although very recent advances (Qiao et al., 2016; Pesaran et al., 2018) have started to make it possible to look at much wider areas. Because of the risks associated with neurosurgery (though see Waldert, 2016) and the ethical issues associated with it, most of the research using microelectrodes has been carried on non-human primates (Taylor et al., 2002; Carmena et al., 2003; Fitzsimmons et al., 2009; Borton et al., 2013) or rats (Chapin et al., 1999). Only much less frequently research has been carried out on humans, mostly on individuals with motor disabilities (Kennedy et al., 2004; Brumberg et al., 2010), and very rarely for cognitive enhancement (Hampson et al., 2018).

### 2.2. Brain Stimulation Technologies

#### 2.2.1. Non-invasive Stimulation Technologies

The most popular non-invasive brain-stimulation technologies are transcranial electrical stimulation (tES), transcranial magnetic stimulation (TMS), and focused ultrasound (FUS).

Stimulating the brain with tES (Nitsche and Paulus, 2000; Moreno-Duarte et al., 2014) involves attaching electrodes to the scalp to inject a small direct (transcranial Direct-Current Stimulation or tDCS) or alternating (transcranial Alternating-Current Stimulation or tACS) current (typically 1–2 mA in intensity) for up to 30 min (for safety reasons – see Parasuraman and McKinley, 2014). Compared to TMS (described below), tES has the advantage of being cheaper and more portable (McKendrick et al., 2015). However, it has the limitation of a poor spatial resolution, although recently higher-definition forms of tES have been developed (Datta et al., 2009; Edwards et al., 2013) and commercialized. Promising results in human augmentation have been obtained with tES (e.g., Clark and Parasuraman, 2014; Coffman et al., 2014), but questions have been raised about its real non-invasiveness (Davis and van Koningsbruggen, 2013), the effects of prolonged use (Wurzman et al., 2016), and the inconsistency in outcome results across different participants (Krause and Cohen Kadosh, 2014). For example, when applying tES to the motor cortex, it seems that only a minority of the participants could benefit from the tES in the form of an increase of motor evoked potentials, suggesting that humans could be divided into “responders” and “non-responders” to tES (López-Alonso et al., 2014). Such significant variability in effects of tES across participants (Horvath et al., 2014) seems to be mainly due to a variety of differences between human brains, including morphological (e.g., head size, tissue thickness) (Datta, 2012) and functional (e.g., different optimal excitation/inhibition balance between brain regions) (Krause et al., 2013; Krause and Cohen Kadosh, 2014).

TMS uses intense electric currents flowing inside a coil placed on the participant's scalp (Pascual-Leone et al., 1995) to create a magnetic field that induces current flows in the underlying cortical tissue altering neural firing (Parasuraman and McKinley, 2014). However, all current TMS designs are limited in many important ways (Epstein, 2014). Firstly, the coils do not allow for very precise focusing of the electromagnetic wave. This results in a resolution of at least 1 cubic centimetre of brain tissue. Secondly, it is impossible to stimulate deeper structures without the concurrent stimulation of shallower ones. Finally, TMS is quite bulky, hence not suitable for mobile applications. Nevertheless, several studies have used TMS for human cognitive enhancement (e.g., Hilgetag et al., 2001; Boggio et al., 2009; Chi et al., 2010; Chi and Snyder, 2012; Manenti et al., 2012) involving a variety of core information processing systems in the brain, such as perception, learning and memory—see the review by Balan et al. (2014) using text mining technology.

With stimulation technologies one may question what their temporal resolution is: Is it the maximum frequency of stimulation or, correspondingly, the minimum period between stimulation pulses? Is it the temporal precision with which a pulse can be delivered? Is it the time between the beginning of the stimulation and the corresponding effects on the brain becoming apparent? With TMS all of these interpretations indicate that the resolution is good. However, for tES the situation is slightly less clear. While it is true that tES can operate in the kHz range, it is typically believed that the effects of the stimulation require some exposure before manifesting themselves. However, there is mounting evidence (e.g., Reinhart and Woodman, 2015) that suggests that tES can provide temporally precise effects on specific functions. Hereafter, we will primarily refer to the delay with which manifest effects on the brain are produced when talking about temporal resolution of stimulation technologies.

FUS is a novel and still experimental transcranial neurostimulation technology that relies on low-intensity focused ultrasound pulsations to produce reversible excitation or inhibition on neurons (see Bystritsky et al., 2011 for a review). Spatial resolution is potentially good (the target can be as small as 1 × 1.5 mm), and also there is no effect on tissues traversed by the beams while converging onto the target position. However, the safety of the procedure is still being investigated and only recently has human experimentation begun (e.g., Lee et al., 2015).

Finally, we should mention electroconvulsive therapy (ECT) (Abrams, 2002)—the administration of a brief-pulse current of about 800 mA delivered using electrodes applied to the temporal lobe for medical purposes. ETC could in principle be considered as a form of cognitive augmentation in that, when used to treat mental disorders, can also indirectly restore to normal cognitive performance affected by the mental disorder. This might potentially happen, for example, in major depression, where cognitive functioning can deteriorate during acute phases (Hammar and Ardal, 2009). Also, though one of the well known side effects of ECT is temporary impairment of cognitive performance, not only the impairments seem to be limited to a few days after ECT, but there are indications that cognitive performance might improve as compared to baseline levels (Semkovska and McLoughlin, 2010). However, we are not aware of any attempt to use ECT for cognitive augmentation applications in healthy participants.

#### 2.2.2. Invasive Stimulation Technologies

Deep brain stimulation (DBS) is an invasive brain-stimulation technology widely used for the treatment of movement (e.g., in Parkinson's disease) and memory disorders. It requires implanting neuro-stimulators in specific parts of the brain, which send electrical pulses to interfere with neural activity at the target sites within the brain. Similarly, implanted electrodes are routinely used in medicine to electrically stimulate focal areas of the brain for the treatment of incoercible epilepsy.

Due to their invasiveness, ethical issues and cost, DBS and implanted electrodes are only used in the medical sector to improve the patients' quality of life. Therefore, cognitive augmentation research on humans with invasive technologies has been so far very limited and carried out with individuals who have implanted devices for other clinical reasons (e.g., Parkinson's disease, epilepsy, etc.). For instance, DBS has been used for learning enhancement (see Clark and Parasuraman, 2014; Suthana and Fried, 2014 for reviews of its applications). Implanted electrodes have been used in visual prostheses, which compensate for a visual sensory loss by coupling a camera to the brain via an electrode array implanted directly on the visual cortex (Dobelle and Mladejovsky, 1974; Dobelle et al., 1979). Recently, intracortical micro-electrode arrays have started to be used to convey information gathered from one rat's brain to another (more on this in section 3.1.4, e.g., Deadwyler et al., 2013; Pais-Vieira et al., 2013) and to improve memory (Hampson et al., 2018) (see also section 3.3).

### 2.3. Comparison of Neuroscience Technologies for Observing and Influencing Brain Activity

**Figure 1:**
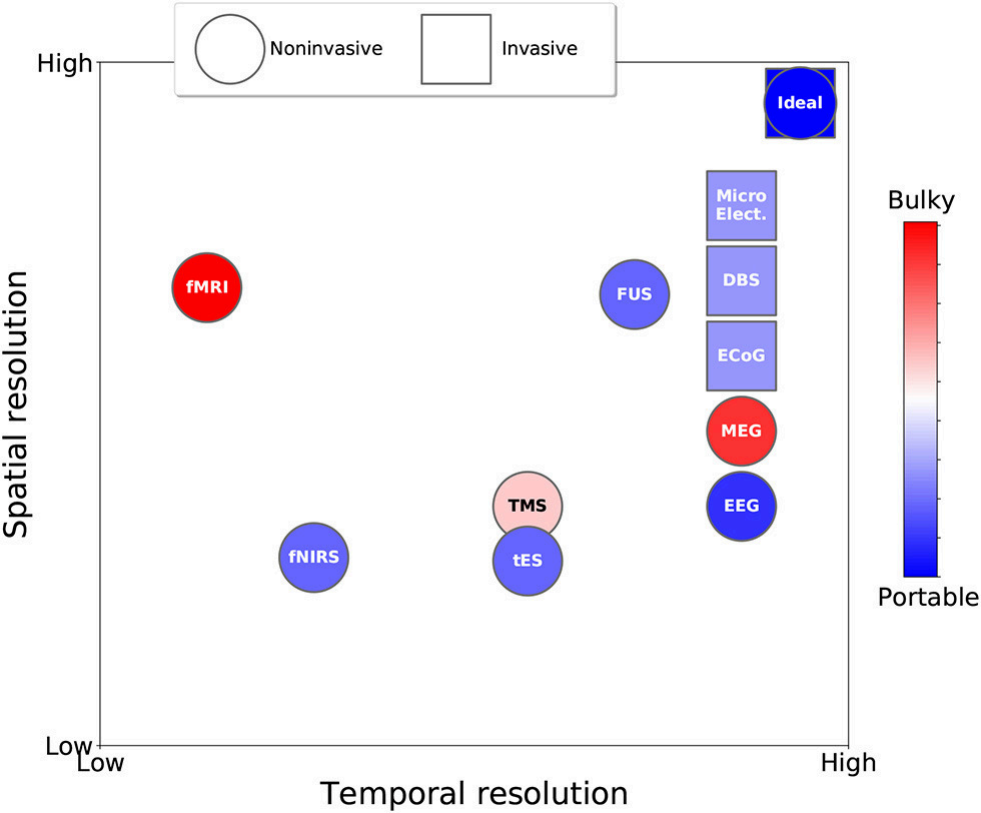
Taxonomy of neuroscience technologies for observing and influencing brain activity based on temporal resolution, spatial resolution, invasiveness (circle vs. square), and portability (color).

Figure 1 shows the trade-offs between spatial and temporal resolution, portability, and invasiveness of the different neuroscience technologies for recording brain activity and for brain stimulation reviewed in the previous sections. Table 1 summarises the main advantages and disadvantages of each technology.

**Table 1:** Advantages and disadvantages of different neuroscience technologies for observing and influencing brain activity.

**Technology**	**Invasive**	**Advantages**	**Disadvantages**
EEG (recording technology)	No	Cheap Portable Very good temporal resolution	Limited spatial resolution Only measures neural activity near the scalp Low signal-to-noise ratio
MEG (recording technology)	No	Good temporal resolution No contact with the body	Expensive Bulky and not portable Primarily sensitive to surface activity Sensitive only to currents in certain directions
fMRI (recording technology)	No	Good spatial resolution No contact with the body	Expensive Bulky and not portable Poor temporal resolution
fNIRS (recording technology)	No	Cheap Portable	Difficult calibration Low spatial and temporal resolution
ECoG (recording technology)	Yes	Good signal quality Good temporal and spatial resolution	Neurosurgery required It only measures neural activity near the surface of the brain Expensive
Implanted micro-electrodes (recording and stimulation technology)	Yes	Good signal quality High temporal and spatial resolution	Neurosurgery required Very limited regions of the brain covered Risks associated to the surgery (e.g., infections)
DBS (stimulation technology)	Yes	It allows the stimulation of deeper brain regions than most other techniques High temporal and spatial resolution	Neuropsychiatric side effects (e.g., apathy) Difficult to keep electrodes in place Risks associated to the surgery (e.g., infections)
tES (stimulation technology)	Yes	Cheap Portable Good spatial resolution for high-definition tES	Low spatial resolution for normal tES Unknown long-term effects
TMS (stimulation technology)	Yes	Good spatial and temporal resolution	Expensive Bulky Unknown long-term effects
FUS (stimulation technology)	Yes	Good temporal and spatial resolution	Insufficiently tested on humans Applicable only to a small area of the brain

As the figure and table indicate, no neuroscience technology for influencing or observing brain activity is optimum. Each technology presents a unique trade-off in terms of spatial resolution, temporal resolution, invasiveness, portability (and indirectly cost). In the figure, the ideal technologies in terms of spatio-temporal resolution are represented by the circle (non-invasive) and square (invasive) symbols in the upper right corner of the plot. Overall, with the exception of FUS, which is still at an experimental stage, non-invasive *stimulation* technologies have lower spatial and temporal resolutions than the best non-invasive brain-activity recording technologies. Also, it can be seen that invasive technologies are closer to the optimum in terms of spatio-temporal resolution than non-invasive technologies, but their widespread adoption is hampered by ethical and medical issues associated with their invasiveness, making them sub-optimal under these other important respects.

An aspect that we have not discussed in our analysis is the power requirements for different technologies. However, as a rule of thumb, wherever we note that a technology requires bulky equipment (red in Figure 1), one can safely infer that power consumption is high (e.g., for fMRI). Conversely, when a technology is classed as portable (blue in Figure 1), it is also battery-powered, implying much lower power consumption.

## 3. Applications of Neuroscience Technologies for Human Augmentation

This section surveys the main applications of neuroscience technologies for human cognitive augmentation. Many of these applications fall into two broad disciplines: *Neuroergonomics* and *Brain-Computer Interfaces* (BCIs). Neuroergonomics examines the neural and cognitive mechanisms underpinning human performance in everyday tasks and in the work place (Parasuraman, 2003; Parasuraman and Rizzo, 2007) and uses such knowledge to design systems that allow humans to perform in a safer and more efficient way. BCIs, instead, have traditionally been more concerned with providing means to compensate for absent or lost functionality in people with severe motor disabilities (Wolpaw et al., 2002; Birbaumer, 2006), allowing them, for example, to control devices such as wheelchairs or computer cursors, or to communicate, when the natural way of communicating is severely lost (Wolpaw et al., 1991; Pfurtscheller et al., 1993; Chapin et al., 1999; Mason and Birch, 2000; Fabiani et al., 2004; Millán et al., 2004; Citi et al., 2008; Huang et al., 2009; Allison et al., 2012a; Yin et al., 2013; Sellers et al., 2014).

In the light of the choice we made in section 1 of embracing a wide definition of human cognitive augmentation that considers augmentation any improvement over the functionality already available in an individual, it is clear that there is a significant overlap between BCIs and Neuroergonomics. The main differences really are the type of users being considered and the application domains of interest for such users. However, even these differences are becoming less and less clear: for instance, neuroergonomics has been applied to rehabilitation (Meinel et al., 2016; Teo et al., 2016; Gramann et al., 2017) and BCIs have been employed to improve decision making in able-bodied individuals (Poli et al., 2014; Valeriani et al., 2017b). Also, as BCI technology continue to develop, BCI spellers and systems for pointer control, that are nowadays only useful for the severely disabled, might become “competitive” with the devices used today by able bodied users. Furthermore, new forms of BCI, namely *passive BCIs* (Zander and Kothe, 2011; Aricò et al., 2017, 2018; Krol et al., 2018), already bridge the gap between neuroergonomics and BCIs by monitoring spontaneous (i.e., not directly triggered by the BCI itself) brain activity of users performing everyday activities, and react in ways that facilitate such activities for the users.

For these reasons, in the following we will not attempt to distinguish between applications developed by neuroergonomics community vs. those developed in BCI, nor will we exclude applications based on the size and nature of their user-base. Instead, as already mentioned, we will focus on the cognitive functions that each application attempts to augment.

The principles of systems for augmenting communication, including brain-to-brain, are presented in section 3.1. Augmentation technologies for cognitive performance and decision-making are considered in detail in section 3.2. Memory enhancement is covered in section 3.3. Attention enhancement and monitoring is discussed in section 3.4. Applications to situation awareness are presented in section 3.5. Hyperscanning and its potential future applications are discussed in section 3.6. Individual differences in the ability to achieve cognitive augmentation and user selection are explored in section 3.7. Personnel training is discussed in section 3.8. Section 3.9 looks at enhancing the ability to solve complex problems.

### 3.1. Communication

BCI systems based on the recording technologies presented in section 2 have typically been used to detect specific (intentionally and unintentionally induced) patterns of brain activity, and translate them into commands for devices or into communication acts (Wolpaw et al., 1991, 2002; Pfurtscheller et al., 1993; Chapin et al., 1999; Mason and Birch, 2000; Fabiani et al., 2004; Millán et al., 2004; Birbaumer, 2006; Citi et al., 2008; Huang et al., 2009; Allison et al., 2012a; Yin et al., 2013; Sellers et al., 2014).

In the following sections we review the main operational principles of the most widespread types of BCIs for communication.

#### 3.1.1. BCIs Based on Event-Related Potentials (ERPs)

Within EEG-based BCIs, those based on ERPs, i.e., series of oscillations in the electrical signal recorded on the scalp in response to suddenly occurring sensory, cognitive, or motor events (Luck, 2014), have been an area of major research activity. In particular, BCI research has focused on the P300 ERP, a large positive peak occurring between 300 and 600 ms after stimulus presentation that is associated with the detection and recognition of interesting, rare, deviant or target stimuli (Polich, 2007). The P300 ERP is especially useful for BCI purposes as its presence depends on whether a user attends to external stimuli.

Based on principles similar to that of the oddball paradigm—where observers are asked to detect a relatively infrequent target stimulus among a sequence of more frequent non-targets (Squires et al., 1975)—P300-based BCIs use a display where different locations are occupied by different stimuli, each associated with a different command. If the stimuli flash in random order and the user only attends to one of them (target), then P300 ERPs are generated only after the flashing of target stimuli and no others. This makes it possible for the BCI to determine which stimulus is being attended to, i.e., which command the user intends to issue. One of the first applications of this type of BCI to communication was pioneered by Farwell and Donchin (1988), who developed a speller based on a matrix of letters flashing randomly at high speed. This inspired the development of a large number of other BCI spellers (see Rezeika et al., 2018 for a review).

Usually, the best P300 recognition accuracy is obtained by temporally spacing the stimuli in such a way that their ERPs minimally overlap. However, with the approaches mentioned above and state-of-the-art machine learning it is possible to build BCIs with high Information Transfer Rates^1^ (ITRs) (Wolpaw et al., 2000) and very short inter-stimulus intervals (e.g., between 100 to 200 ms). Fast stimulus presentation is, therefore, routinely used in modern BCIs.

An advantage of P300-based BCIs is that they require minimum or no prior user training. A disadvantage is that, despite the P300 being the largest ERPs, single instances of P300s are still difficult to detect reliably. For this reason, in some P300-based BCIs users are required to issue the same command a number of times (e.g., 3–5) to achieve higher accuracy. This, of course, slows down the issuing of commands (and, correspondingly, the ITR of the BCI) and can limit the usability of the BCI.

#### 3.1.2. Other Forms of EEG-Based BCI for Communication

Other EEG-based BCIs for communication are based on different types of neural activity. Amongst those more frequently used are Slow Cortical Potentials (SCPs), Mu Event-Related Desynchronization (ERD), mental imagery, and Steady-State Visually Evoked Potentials (SSVEPs)—which instead depends on external stimuli. SCPs, ERD, and mental-imagery BCIs are fundamentally based on biofeedback principles (see for example Birbaumer et al., 1981) and are not dependent on external stimuli, in the way in which ERPs-based and SSVEP BCIs are. For this reason, they are typically classed as *self-paced* BCIs.

SCPs consist of slow shifts in the EEG produced over large portions of the scalp. Through extensive training, individuals can learn to voluntarily produce positive or negative SCPs in the EEG. This can be achieved with emotional or mental imagery, which may generate some weak SCPs, but later the generation of SCPs becomes automatic. A BCI can then recognize the positive SCPs from the negative ones and from the no-SCP state, and then convert them into commands for an external device, for example a speller (Kotchoubey et al., 1997; Birbaumer et al., 1999; Kübler et al., 1999, 2001; Birbaumer, 2006). Given the time and effort required to operate these BCIs and their relatively poor performance, SCP-based BCIs are often used only with locked-in patients.

ERD-based BCIs exploit the Mu (or sensorimotor) rhythm, which presents itself as oscillations in the frequency range 8–12 Hz and is associated to movement planning and execution. The rhythm attenuates with movement (or imaginary movement) of specific parts of the body, due to the corresponding area of the brain becoming more active (and the corresponding desynchronization of neuronal activity). Movements of the right part of the body desynchronize Mu activity in the left hemisphere of the brain, and *vice versa*. Left and right ERDs can, therefore, be recognized and interpreted as two distinct commands by a BCI, which can be used to control a spelling device (Pfurtscheller and Neuper, 1997, 2006; Scherer et al., 2004). Initially, mu activity is voluntarily modulated by movement-related imagery (e.g., imagining hand or foot movements). However, through training and real-time feedback about the intensity of their own mu activity, users can learn to directly produce mu rhythms of varying intensities and locations without the need to use any specific mental task. Evidence suggests that only a relatively small portion of participants can achieve high levels of performance, with some being completely unable to control mu rhythms.

SSVEPs are involuntarily generated in the brain when the retina is excited by a visual flashing stimulus of a particular frequency (typically in the range of 4–40 Hz). This oscillatory activity can easily be recognized by the BCI via a simple frequency analysis. Typically, SSVEP-based BCIs use a display containing multiple stimuli (each representing a different command) flashing at different frequencies (Amiri et al., 2013). If users can control their gaze, then they can simply direct it to individual flashing stimuli, thereby producing SSVEPs of distinct frequencies. This allows the BCI to recognise the command a user intends to issue and can, therefore, be used for communication (Cecotti, 2010; Hwang et al., 2012; Yin et al., 2015). Research has shown that it is not always necessary to have gaze control: in some SSVEP-based BCIs it is sufficient to shift one's attention to one of the flashing stimuli (Lopez-Gordo et al., 2010; Allison et al., 2012b; Amiri et al., 2013).

#### 3.1.3. Invasive BCIs for Communication

Invasive recording technologies have been used in some forms of augmentation technologies which, unsurprisingly, thanks to the better-quality brain signals recorded, have better performance/ITR than corresponding non-invasive ones (Tehovnik et al., 2013; Baranauskas, 2014). Of course, due to potential medical and ethical problems associated with electrode implantation, most of the research on invasive BCIs has been carried out with monkeys (Taylor et al., 2002; Carmena et al., 2003; Fitzsimmons et al., 2009; Borton et al., 2013) or rats (Chapin et al., 1999), and only less frequently humans. In this section we will only focus on work for human communication augmentation.

Versions of the matrix speller discussed in section 3.1.1 based on ECoG (see section 2.1.2) have been developed (Leuthardt et al., 2006; Brunner et al., 2011; Krusienski and Shih, 2011; Zhang et al., 2013). These have shown promising results (particularly Brunner et al., 2011, which achieved a peak ITR of over 100 bits/min). However, only patients who need to have ECoG implanted for medical reasons could benefit from this technology.

Other invasive BCIs for spelling are based on the selection of letters from an on-screen virtual keyboard using 2–D pointer control. For instance, in Kennedy et al. (2000, 2004) a cortically-implanted glass microelectrode filled with a neurotrophic growth factor was used to record local field potentials in amyotrophic lateral sclerosis patients, while in Bacher et al. (2015) a 96 micro-electrode array was implanted in a tetraplegic patient who was able to input up to 10 correct characters per minute.

BCIs based on implanted electrodes have also been used to provide speech, rather than written text, capabilities to the paralyzed. In this context, the BCI is used to *predict intended speech information* directly from the activity of neurons. Such information is then used to directly control a speech synthesizer (Brumberg et al., 2009, 2010; Guenther et al., 2009). Users have nearly instantaneous feedback, which makes it possible for them to improve their speech synthesis over time. However, in those studies only a limited range of speech acts was possible. In tests with one patient, vowel production was achieved “with reasonably high accuracy, attaining 70% correct production on average after approximately 15–20 practice attempts per session” (Brumberg et al., 2010). Fortunately, more recent work (Herff et al., 2015) has significantly improved the performance of such systems by combining BCIs and speech-recognition technology. This hybrid approach achieved, in the best conditions, word error rates as low as 25% for a dictionary of 10 words.

#### 3.1.4. Brain-to-Brain Communication

Recently, researchers have started exploring the possibility of *brain-to-brain communication*, i.e., physically and directly connecting brains for the purpose of allowing direct exchanges of information. This was first theoretically proposed by Nicolelis (2011) and was successfully tested in Pais-Vieira et al. (2013, 2015) in rats, where an encoder rat was trained to perform a task that was then “communicated” to a decoder rat. More specifically, the synaptic activity in the motor cortex of the encoder rat was invasively recorded while performing one of two different tasks, and transmitted to the decoder rat with invasive intracortical micro stimulation. This allowed the decoder rat to learn to perform the same task. In a similar manner, memory or acquired knowledge was transmitted via brain-to-brain communication by Deadwyler et al. (2013), where hippocampal activity of donor rats associated with short-term memory tasks was transmitted to the brains of naive receiver rats improving their task performance.

The first *non-invasive* system for brain-to-brain communication was proposed by Yoo et al. (2013), who used an SSVEP-based BCI to recognize when a *human* participant wanted to stimulate a rat's tail movement, and delivered the command to the rat's brain via a transcranial ultrasound burst (FUS, see section 2.2.1), which stimulated the motor cortex of the rat, triggering a tail movement. Non-invasive brain-to-brain communication has also been achieved with humans, for example, in Grau et al. (2014) where a motor-imagery-based BCI was used to produce binary-encoded words, which were then transmitted to a receiver in the form of phosphenes induced via TMS burst. In other recent studies (Rao et al., 2014; Jiang et al., 2018), brain-to-brain communication has been used to transmit information between individuals in a collaborative task, again by combining EEG and TMS. In Jiang et al. (2018), for example, groups of three individuals collaborated to accomplish a Tetris-like game. In that case, two senders transmitted information remotely about whether to rotate a block to a receiver who was conveyed the information via TMS on the occipital lobe. The receiver integrated the information and actuated his/her decision about whether to rotate or not the block via EEG. In Stocco et al. (2015) pairs of senders and receivers collaborated bi-directionally in a question-and-answer task.

Of course, all of the above mentioned studies present a number of limitations, including the fact that the communication is restricted to very limited type of information, and that the ITR is very low (for a discussion of some of the limits see for example Stocco et al., 2015). However, as for other neurotechnologies for cognitive augmentation, the achievements so far in brain-to-brain communication represent an important proof-of-concept, and its development might potentially lead to future systems that outperform or complement natural ways of communication (such as talking).

### 3.2. Cognitive Enhancement

This section presents work that has been carried out in recent years to develop neurotechnologies that can enhance cognitive abilities, with a focus on BCI applications for individual (section 3.2.1) and collaborative (section 3.2.2) decision making, and cognitive enhancement based on brain stimulation (section 3.2.3).

#### 3.2.1. Individual Decision Making

Decision-making has been intensively studied in social and cognitive sciences to understand the processes, dynamics, biases, and strategies that lead to optimal decisions (Edwards, 1954; Janis and Mann, 1977; Sniezek, 1992; Plous, 1993; Cannon-Bowers and Salas, 1998), both when made by an individual or a group. A decision is affected by, and is the result of, a number of processes and mechanisms that include—but are not limited to—early perceptual processes, attention and working memory processing, all of which are critical to an optimal decision.

Advances in neuroscience have provided a deeper understanding of neural processes related to decision-making. For example, the amplitude of the N1—a large negative ERP occurring between 80 and 120 ms after the onset of an unpredictable stimulus in the absence of task demands—decreases as the attentional level decreases (Parasuraman and Beatty, 1980; Parasuraman et al., 1982; Hillyard and Anllo-Vento, 1998; Luck et al., 2000), while its timing is sensitive to the difficulty of the task. The difficulty of a task also affects amplitude and timing of the P300 (Hagen et al., 2006; Luck, 2014). These ERPs are typically associated with early perceptual and cognitive processing of events, and can reveal fatigue in perceptual decision-making. For instance, this is signaled by a reduction of the amplitude and an increase of the latency of the P300 (Uetake and Murata, 2000; Murata et al., 2005).

Other, later ERPs are instead associated with decision processes preceding, for example, the overt response of a decision maker. For instance, the *contingent negative variation*—a slow negative wave related to the preparation for a motor response and stimulus anticipation—is smaller before incorrect responses than before correct ones in a task where information necessary to identify a target letter is conveyed to participants only a few hundred milliseconds before two potential targets are presented (Padilla et al., 2006). This ERP can be used as a basis for detecting decision-making cheats/lies (Fang et al., 2003) or to decide whether a driver wants to accelerate or pull the brake (Khaliliardali et al., 2012).

The *error related negativity*—an ERP occurring 50–80 ms after an incorrect response—can also provide information about levels of confidence of decision-making as it is affected by confidence in own performance (Selimbeyoglu et al., 2012). This happens even when participants are unaware of the error (Nieuwenhuis et al., 2001). Moreover, neural correlates of individual decisions can be detected hundreds of milliseconds before an explicit response is given—(e.g., Tzovara et al., 2012). Error related negativity can also be used to automatically improve the speed of communication in BCI spellers (Dal Seno et al., 2010; Schmidt et al., 2012; Spüler et al., 2012), or to identify decision errors in a forced-choice task under time pressure (Parra et al., 2003).

Recent advances in neuroscience have also shed light on how individuals approach decision-making, their strategies and their aptitude to risk-taking behavior (Doya, 2008; Rushworth and Behrens, 2008). For example, there is evidence showing a large involvement of the prefrontal cortex in decision-making; in particular, its activation varies according to the level of risk taking (Tobler et al., 2009). However, to date this knowledge has not been exploited for human augmentation.

Thanks to this plethora of neuro-scientific knowledge related to information and decision processing, it would seem reasonable to attempt exploiting it to improve decision-making. However, the most practical non-invasive sources of information on brain activity are extremely noisy, which makes it very hard to reliably provide information on (or aid) individual decisions. Indeed, the aforementioned reports base their findings on *averaging* the signals resulting from many repetitions of each event. As shown in the next section, this limitation can be overcome if during the decision making process information is gathered from multiple brains.

#### 3.2.2. Group Decision Making and Collaborative BCIs (cBCIs)

In the last few years, researchers have started evaluating the possibilities offered by ERP-based single-trial *collaborative* BCIs. These integrate perceptual experiences, intentions and decisions from multiple non-communicating users to achieve improved joint performance over single-user BCIs and non-BCI systems.

Various methods can be used to integrate EEG data from multiple participants (Wang and Jung, 2011; Stoica, 2012). Raw signals can be averaged across participants in order to build a sort of “group EEG.” The resulting signals can then be processed by a single BCI. Alternatively, one can first extract meaningful features from the EEG data of each participant and then concatenate them to build a feature vector for the group, which is then passed to a single classifier. Finally, users may have individual BCIs that predict their intentions, which a voting system integrates to compute the group's decision. Various studies (Wang and Jung, 2011; Matran-Fernandez et al., 2013; Stoica et al., 2013; Jiang et al., 2015) suggest that this voting method is often optimal for collaborative EEG-based classification, especially when the scores of the single classifiers (instead of the predicted class) are used for the integration (Cecotti and Rivet, 2014). Wang et al. (2011) proposed a first collaborative framework for BCIs where an ensemble classifier was used to integrate the outputs of single BCIs. They showed that collaborative BCIs could improve the classification rate in a visual target-detection task from 69% (individual performance) up to 99% for groups of 20 participants. Later, they showed that cBCIs could also predict movement directions better and faster than single-user BCIs (Wang and Jung, 2011), but never better than a single non-BCI user. In Yuan et al. (2012), a proof-of-concept cBCI for detecting the onset of visual stimuli presented on a black background was proposed. The stimuli produced visually evoked potentials that the cBCI could detect more accurately than a single-user BCI. Decisions were faster, but accuracy was substantially lower, than for non-BCI users.

Eckstein et al. (2012) investigated voting methods for integrating single BCI outputs to improve performance in a decision task where observers had to discriminate between faces and cars. They found that cBCIs not only improve accuracy, but can also make the decisions faster than the average human. However, at least seven individuals were required to achieve the behavioral performance of the average single observer. Yuan et al. (2013) used approximately the same experiment with a cBCI which detected target stimuli more accurately than a single-user BCI and responded faster than non-BCI users, but with substantially lower accuracy. Cecotti and Rivet (2014) found that combining data from multiple participants provides more advantages in terms of accuracy than combining data from the same participant over time. Moreover, they showed that with the collaborative approach every group member makes a contribution to the overall performance of the group.

A different approach has been used by Poli et al. (2014), who developed a hybrid cBCI that integrates behavioral and neural data to achieve group decisions that are better than both the average single observer and traditional non-BCI groups. Instead of predicting the user's response, this cBCI used neural signals and response times to estimate the decision confidence group members and weigh their behavioral responses accordingly to build the group decision. This paradigm was tested with various tasks, including visual matching (Poli et al., 2014), visual search with simple shapes (Valeriani et al., 2015b, 2017c), visual search with realistic stimuli (Valeriani et al., 2015a, 2017b), face recognition (Valeriani et al., 2017a), and threat detection with video stimuli (Valeriani et al., 2018). In all cases, it was found that the cBCI reduced error rates by up to a third with groups of only two users when compared with traditional equally-sized non-BCI groups using the standard majority, indicating that hybrid cBCIs for decision-making are promising.

cBCIs have also been applied in other contexts partly related to decision-making, including face recognition (Jiang et al., 2015; Valeriani et al., 2017b), target detection (Matran-Fernandez et al., 2013; Stoica et al., 2013), and localization (Matran-Fernandez and Poli, 2014, 2017b). For instance, Matran-Fernandez et al. (2013) used the presence of P300s to detect aeroplanes in rapidly presented aerial pictures. The N2pc, an ERP that appears approximately 250 ms after stimulus presentation on the opposite side of the scalp with respect to the visual hemispace where an object of interest is located, has been used for BCIs for determining the location(rather than the presence) of targets in aerial pictures (Matran-Fernandez and Poli, 2017a).

Collaborative BCIs have also been used to control robots (Iturrate et al., 2013; Katyal et al., 2014; Li and Nam, 2015), video games (Nijholt and Gürkök, 2013; Nijholt, 2015), cursors and simulated space crafts (Poli et al., 2013), spellers (https://www.youtube.com/watch?v=A3SnmhlOTtQ) as well as to analyse the neural signals of people watching movies and identify a relationship between the length of a shot and the amplitude of a large-scale ERPs called post-cut negativity (Matran-Fernandez and Poli, 2015). For a review on collaborative BCIs see (Valeriani and Matran-Fernandez, 2018).

#### 3.2.3. Brain Stimulation for Cognitive Enhancement

Neuro-stimulation techniques, such as tES and TMS, can be used to improve performance in different cognitive domains, including perception, learning and memory, attention and decision making (some of which will be reviewed in sections 3.3 and 3.4; for a review, see Coffman et al., 2014).

Several studies have shown how the ability to detect (e.g., via visual search) or track specific targets can be improved. For example, performance in visual search was improved in a tDCS study (Nelson et al., 2015), where observers were presented with a display containing simple, colored shapes and had to decide whether a target was present or not. Results showed that anodal stimulation slightly improved performance. Similar results were obtained in a more realistic, complex threat-detection task with tES (Clark et al., 2012). In that study, observers were presented with a short video clip recorded from a virtual reality environment and had to decide whether a possible threat was present or not. In the two experiments conducted in the study, the use of tES significantly and consistently improved performance. Multiple object tracking is another task often associated with (and preceding) complex decision-making in many situations and where tES can augment human abilities. In Blumberg et al. (2015), participants were asked to focus their attention on two (low-load) or four (high-load) particular circles (targets) out of the eight displayed. The circles were then moved around for 8 s and then participants were asked to manually select which circles were the target. This required users to track multiple moving objects. Results indicated that tES significantly improved performance of participants in the high-load condition, but only marginally improved performance in the low-load condition.

Risk-taking behavior can also be affected by tES. In particular, Sela et al. (2012) have shown that left stimulation of the dorsolateral pre-frontal cortex (DLPFC)—an area that is known to be involved the process of evaluating risks and benefits—resulted in participants exhibiting a much riskier decision-making behavior than participants receiving right hemisphere or sham stimulation. Another study, however, has shown that concurrent anodal tDCS of the right DLPFC and cathodal tDCS of the left DLPCF can diminish risk-taking behavior (Fecteau et al., 2007).

tDCS has also been used to treat reading disabilities like dyslexia, showing promising results in both adults (Heth and Lavidor, 2015) and children (Costanzo et al., 2016). However, the improvement in reading brought by tDCS seems only to apply to certain tasks, such as sight word efficiency (Younger et al., 2016).

Finally, brain stimulation could also be used to optimize cortical oscillations (e.g., alpha and theta), which in turn may indirectly lead to enhancements in several tasks (e.g., stimulus binding) (Horschig et al., 2014).

### 3.3. Memory Enhancement

The use of non-invasive stimulation with TMS and tES has been shown to improve memory and learning in a large number of studies (for reviews/meta-analyses, see Brunoni and Vanderhasselt, 2014; Madan, 2014). For example, tDCS stimulation has been observed to improve: implicit learning of sequential motor sequences (Nitsche et al., 2003; Reis et al., 2009), complex forms of motor learning (Hunter et al., 2009), implicit probabilistic learning (Kincses et al., 2004), explicit memory for lists of words (Hammer et al., 2011), spatial memory (Flöel et al., 2012; Foroughi et al., 2015) and working memory (e.g., via the N-Back and Sternberg tasks) both in healthy individuals and individuals with memory deficits (Fregni et al., 2005; Bennabi et al., 2014; Brunoni and Vanderhasselt, 2014). In these studies, particularly effective seems to be the stimulation of the dorsolateral prefrontal cortex, which is known to be a critical locus for working memory functions (Levy and Goldman-Rakic, 2000).

In relation to the duration of the benefits of tES/TMS stimulation on short- and long-term memory, a number of studies suggest that these can persist for up to 4–6 weeks after stimulation (Ohn et al., 2008; Lally et al., 2013; Myczkowski et al., 2018). However, the evidence is mixed (Teo et al., 2011; Brunoni and Vanderhasselt, 2014).

Studies with invasive stimulation neurotechnologies have also shown promising results. Recent successes include the development of neuroprosthesis that can improve memory encoding and retention. These are based on a nonlinear systems approach that computes multiple-input/multiple-output (MIMO) associations, where inputs are spike trains from neurons in the hippocampus area CA3 generating output spike trains in the area CA1(Berger et al., 2005, see also Berger et al., 2010, Figure 5, Berger et al., 2011, Figure 2, and Madan, 2014, Figure 1 for schematic representations of the MIMO model). The two areas are both crucial in the formation of memories, particularly for the “transition” of memory contents from short- to long-term memory. The neuroprostheses have demonstrated that real-time manipulation of the encoding process can restore and even enhance mnemonic processes in rodents (Berger et al., 2011) and non-human primates (Hampson et al., 2013). In particular, the pattern of activation predicted by the MIMO model from the activation of the neurons in CA3 is artificially applied via electrical stimulation to neurons in area CA1. The application of the model in rats' hippocampus has allowed the transference of memories between animals (Deadwyler et al., 2013). More recently, the first successful implementation of the neuroprosthesis, based on the MIMO model, in human subjects has been demonstrated (Hampson et al., 2018). In the study, short- and long-term memories in a delayed match-to-sample task were improved by 37 and 35%, respectively.

DBS in the hippocampus and the entorhinal cortex has also been successful at improving memory (Hamani et al., 2008; Suthana et al., 2012; Suthana and Fried, 2014).

### 3.4. Attention Monitoring and Enhancement

An increasing number of studies and technologies are aimed at monitoring cognitive performance and capacity, for example working memory capacity or attention, in real time (Durantin et al., 2015). Even when such systems are not directly aimed at augmenting performance, monitoring the mental state of users makes it possible to enhance their performance by adapting the interface they interact with, with so called *adaptive interfaces*. For instance, Wilson and Russell (2007) described a neuroadaptive system where the users' task is to detect a target in an environment and where the mental workload is varied according to the feedback given by EEG and other physiological measures. So, many of the studies described below may enable indirect human cognitive augmentation.

There is a vast literature on methods for monitoring changes in the level of attention. In general, the literature makes a distinction between *vigilance* (i.e., the ability of maintaining sustained attention) and the ability to maintain attention in situations of *high workload*, which typically require high involvement of working memory, and the ability to shift, control or divide attention (Parasuraman, 1984). Thus, vigilance means a sustained efficient conscious “detection or discrimination of stimuli, including a simple cognitive or motor response but excluding ‘higher’ attentional or executive functions such as spatial orienting, resolving interference, dividing attention, or selecting between several overt responses” (Langner and Eickhoff, 2013).

Tasks used to monitor vigilant attention include simple reaction-time tasks, stimulus-discrimination tasks and target counting. In all these cases vigilance is gauged using reaction times. Apart from the type of task, the duration of sustained attention without breaks is a major determinant of performance (Davies and Parasuraman, 1982).

Overall, low-frequency EEG rhythms and ERP amplitudes increase as vigilance decreases (Pfurtscheller and Aranibar, 1977). Changes in patterns of EEG activity that accompany the awake-sleep transition can also reveal decreases in attention (see Oken et al., 2006). The most consistent of such measures are an increased theta activity and decreased beta activity (Belyavin and Wright, 1987; Parasuraman and Rizzo, 2007). The amplitude of the P300 ERP is also known to be related to the mental workload and the level of attention devoted to a task. This has also been examined in complex flight and driving simulation tasks where it has been shown that the P300 can provide an assessment of workload (Fu and Parasuraman, 2007). Other, earlier, ERPs can be modulated by attentional allocation. For example, it is known that the N1 amplitude is modulated by allocation of attention to both visual and auditory stimuli in high-load conditions (Hink et al., 1977; Parasuraman, 1978, 1985).

Research on sustained attention/vigilance focuses on tasks that are cognitively undemanding, where the purpose is examining the cognitive and neural process underlying constant vigilance. These are different from the processes where the cognitive load is high, and attention has to be maintained in order to process all the information needed to perform correctly a given, often demanding, task. This ability is often investigated in tasks where there are high demands on working memory. For example, in Gevins and Smith (2007) participants were asked to perform a task consisting of viewing a continuous sequence of stimuli and having to indicate when the current stimulus matches the one from *n* steps earlier in the sequence while EEG was recorded. It was found that as the difficulty of the task (*n*) increased, there was a corresponding increase in theta rhythm and a decrease in the alpha rhythm around the anterior-midline cortex.

Given that changes in attention correspond to specific, detectable patterns of EEG activity, over the years, scientists have tried to developed methods—and applications—to monitor sustained attention and the ability to respond to high workload. For example, methods have been developed to detect drowsiness (see Gevins and Smith, 2003), based on the amplitude of different rhythms, in tasks similar to those one might face in real-world environments. Recent applications in real operational environments include monitoring the mental workload for air traffic controllers during realistic control tasks (Aricò et al., 2016) and the annotation of targets of interest in full-motion video in Army-relevant scenarios (McDermott et al., 2015).

Studies using transcranial Doppler echography and fNIRS also suggest that temporal variations in vigilance and changes in mental workload are accompanied by variations in the cerebral blood flow (e.g., Hitchcock et al., 2003). They also suggest a critical role of the right parietal lobe in the control of vigilance as also seen in the EEG studies discussed above. Changes in mental workload can also be monitored by measuring cerebral hemodynamic changes using fNIRS in real-world environments (Ayaz et al., 2013).

Some research has also been devoted to decoding the spatial orienting of attention (Astrand et al., 2014), with several recent studies showing that monitoring the location of attention—for example, left/right or up/down locations—in real time is possible, with the use of EEG (van Gerven and Jensen, 2009; Treder et al., 2011), NIRS (Morioka et al., 2014), and fMRI (Andersson et al., 2011, 2012).

Reliable monitoring of attention and vigilance allows to identify when it is time to reduce tasks related demands on users, by slowing down the task, removing distractions, or simply asking users to take a break, all of which would lead to an overall cognitive performance advantage over the cases where all is fixed. Of course, brain-stimulation technologies can also be helpful, as they have experimentally been proven to enhance attention. For instance, a repetitive form of TMS was shown to enhance visual spatial attention on the opposite side of stimulation (Hilgetag et al., 2001). Also, there is a significant literature on enhancing different aspects of attention using tDCS (see Coffman et al., 2014 for a recent review). For example, Nelson et al. (2014) performed tDCS on users engaged in a simulated air traffic control task, and found that, while performance in the sham condition deteriorated, as expected, with time, in the active tDCS condition there was a overall improvement in terms of target detection. Other studies have found effect of tDCS in the orienting of attention (Stone and Tesche, 2009), while Gladwin et al. (2012) found beneficial effects of tDCS on selective attention when users where performing a Sterneberg task.

The possibility of enhancing visual attention through the use of BCIs as a mechanism of neurofeedback has also been explored (Lim et al., 2010; Ordikhani-Seyedlar et al., 2016; Strehl et al., 2017) although its efficacy has only been tested on patients with ADHD. Neurofeedback has also been shown to be effective at training tinnitus patients to control their attention to the auditory perceptual modality (thereby giving them the ability to suppress or reduce the effects of tinnitus) (Busse et al., 2008).

The possibility of building passive BCIs that monitor cognitive load in pilots in real flight conditions has been recently demonstrated (Gateau et al., 2018).

### 3.5. Situation Awareness

*Situation awareness* refers to the perception, knowledge and understanding of the status of complex, dynamic scenarios at any particular point in time. Situation awareness is not about general cognizance, but about being aware of what is happening that is relevant for a specific task or goal at hand (Endsley, 1995).

Over several decades, a great deal of research has been conducted to understand all the different aspects of situation awareness, and many different models have been developed (e.g., see reviews in Lau et al., 2013; Lundberg, 2015). Situation awareness consists of three levels of ability (Endsley, 1995): Level 1, the perception of elements or cues in the environment; Level 2, the integration of what is perceived and the understanding of what that means in a particular context; and Level 3, understanding/predicting what may happen within a situation of future based on current knowledge. The study of situation awareness, for example, can be applied to military command and control and combat aircraft, air traffic control, emergency services and a variety of other domains where the information load and flow can be high and mistakes can have disastrous consequences.

From a point of view of cognitive processing, situation awareness includes a large number of factors, with perhaps the most critical ones being attention and working memory. Their relevance for situation awareness has been highlighted in a number of studies (for example, Jones and Endsley, 1996; Durso and Gronlund, 1999).

Recent studies have shown the possibility of using neurophysiological methods to assess the cognitive processes associated with situation awareness in experiments based on simulations of military situations (Berka et al., 2005, 2006). In Catherwood et al. (2014), Level-1 situation awareness was quantified from brain activity recorded with 128-channel EEG in two tasks: one requiring the identification of a target and another identification of threats in urban scenes. In both, the target was changed without warning, producing a loss of situation awareness. It was found that there is co-activity in visual regions and prefrontal, anterior cingulate and parietal regions linked to cognition under uncertainty in the 100–150 ms following the loss of situation awareness. As illustrated in Yeo et al. (2017), situation awareness can also be monitored in air-traffic controllers in real-time and accurately with portable EEG equipment.

Compared to standard measures that solely rely on behavioral outcomes (i.e., task performance) and/or self- or observer-based assessments, neurophysiological methods open up the possibility of developing real-time attention and situation awareness monitors that could be used within a closed-loop/passive-BCI system. For example, Abbass et al. (2014) have recently developed a system to monitor situation awareness in air traffic controllers, where the theta and beta EEG rhythm ratio was used as a measure to assess the workload and the information system *adapted in real-time to make it easier for the controller to cope with the task*.

### 3.6. Social Interactions and Hyperscanning

*Hyperscanning* refers to a technique where the neural activity of two or more individuals, who are engaged and interacting in a common task, is simultaneously recorded (see Babiloni and Astolfi, 2014 for a recent review). Currently hyperscanning is mostly used to identify correlations in the brain activity of interacting individuals. Typical tasks are from the field of game theory, where the consequences of a player's choice also depend on the (unknown) behavior of other interacting players, as in the “Prisoner Dilemma” or the “Trust Game.” Studies using hyperscanning have identified some of the neural correlates of the interaction in two brains, and have documented how these change as the players get to know each other and their interaction during the game evolves as do, for example, their mutual trust (King-Casas et al., 2005; Tomlin et al., 2006), their level of cooperation/competition and the chance of their defecting (Babiloni et al., 2007; Astolfi et al., 2010; Cui et al., 2012). In De Vico Fallani et al. (2010) hyperscanning on individuals playing an iterated version of the Prisoner's Dilemma made it possible to predict non-cooperative interactions with 91% accuracy based on the neural activity recorded in the four seconds preceding their taking place.

At present hyperscanning is not used as yet for communication, cognitive enhancement or to enhance social interactions (such as those occurring in collaborative problems-solving and decision-making). However, in the near future this technique promises to deliver enhancements to such activities.

### 3.7. Individual Differences in Human Cognitive Augmentation and Participant Selection

Given individual differences in cognitive functions and job performance and their interrelation with personality traits (Barrick and Mount, 1991), personnel selection is often based on personality tests in many domains (Cook, 2008), including management, sales, clerks, policing, firefighting, vehicle operators, and so on. For example, in the military context, personnel selection has a long history (Rumsey, 2012) and over several decades, tests of all sorts and behavioral analyses have extensively been used to assess, for example, personality (e.g., Stark et al., 2014), how fast individuals learn, their psychomotor skills, their attitude to risk and their behavior in the face of uncertainties. In domains where specific tests that can predict performance are not available, selection of personnel can still be done based on performance on the job, prior performance on closely related tasks or performance during training for a job.

It is clear that BCIs, brain stimulation and other neuroscience technologies for human augmentation provide *individual-dependent benefits*. For instance, in tES technologies, there is a marked variability in individual responses to the stimulation, some people being cognitively impaired by the stimulation, rather than cognitively augmented (e.g., Sparing et al., 2008; Wiethoff et al., 2014). Also, not every user is able to control a BCI system to an acceptable level—a property called *BCI literacy* (Kübler and Muller-Putz, 2007). For instance, in SCP-based BCIs, even after weeks of training only about 70–75% of people can learn to achieve satisfactory performance. This proportion is higher in BCI based on ERPs (e.g., P300) which typically can be used since the first sessions and where satisfactory control can be achieved by about 80% of users (Kübler and Muller-Putz, 2007; Guger et al., 2009; Cipresso et al., 2012). For ERP-based BCIs, BCI literacy mainly depends on individual differences in the brain activity produced in response to external stimuli (Polich, 1997) (some people, for example, will produce P300 ERPs not large enough to be reliably detected in EEG recordings). However, the successful use of SCP-based BCIs depends on more complex factors, including the ability of a user to learn to voluntarily control brain activity.

Recent advances in neuroscience (such as those in decision-making, mentioned in section 3.2) have brought about also a new possibility: individual differences and specific abilities could be detected not only by measuring behavioral features, but also through the characterization of brain activity. For example, prefrontal cortex activation during decision-making varies according to risk-taking propensity (Tobler et al., 2009). Also, there are indications that visual working memory capacity might be predicted by neural activity in, for example, the prefrontal and parietal cortex, and the basal ganglia (Luck and Vogel, 2013) (see also section 3.3). This type of finding opens up the possibility of using neuro-screening in the future as an effective strategy for personnel selection.

Irrespectively of how benefits of augmentation technologies are assessed, it is clear that there is divide between those who can benefit from human cognitive augmentation technologies and those who cannot. Naturally, what matters is the performance before and after a technology for human augmentation is applied. So, for someone locked-in, a speller with an ITR of 20 bits/min would provide an significant level of augmentation, while for an able-bodied person it would be intolerably slow compared with a keyboard. However, also within particular user-groups which may benefit on average, currently human cognitive augmentation is not for every one. Also, even when there is augmentation, the improvement may be too small to be worth the effort/cost/risk/time.

Beyond this level of selection, if the human cognitive augmentation is to be provided to allow individuals to perform specific, e.g., high-responsibility, jobs, then it is natural that some additional form of selection based on performance will be applied. Interestingly, the performance of interest is that *with* the augmentation technology in action. Because the benefits it provides vary significantly from person to person, this may mean that a person who is best without the technology may not the best when this is activated.

Finally, we should note that in addition to selection based on individual performance (with/without augmentation technologies), abilities, characteristics and physiological measures, user selection can also be based on the contribution to group's performance. For example, in a study of collaborative BCI applied to target detection in rapidly presented streams of aerial images, Matran-Fernandez and Poli (2014) found that performance of the collaborative BCI further improved when members of the group were selected based on the “similarity” in individual performance. In other words, the performance of a BCI-assisted group improved the most when the levels of accuracy in the task of its members were similar. This is also confirmed by other research in group decision-making showing how group performance can depend on group composition, particularly similarity or familiarity between members (Hinds et al., 2000).

### 3.8. Personnel Training

The idea of using neuroscience technologies for personnel training has recently attracted significant interest in the security and defence domains (Stanney et al., 2011; Behneman et al., 2012; Miranda et al., 2014). In particular, these technologies could potentially speedup and improve training—thereby augmenting the abilities of the trainee—by making it possible to meaningfully adapt the training to the users instead of using a more traditional one-size-fits-all approach.

Brain-activity recording technologies can be used to improve training. For instance, Miranda et al. (2014) used EEG-based and other physiological correlates of task learning to improve an individual's learning rate. In the study, a closed-loop system was developed that provided continuous physiological monitoring and feedback (visual, auditory, or haptic) to the trainee in real-time, accelerating learning during sniper training and decision-making (Behneman et al., 2012). EEG can also be used to assess and maximize the outcome of cognitive training interventions, where learners repeatedly perform cognitive tasks to improve their cognitive abilities (Taya et al., 2015). One of the very few MEG-based BCIs in the area of training have been described by Mellinger et al. (2007), who show how MEG can help people learn to modulate their brain signals which, in turn, helps with BCI control.

Neurostimulation techniques, such as tES, can also be used to improve task learning in visual search and exploration (Bolognini et al., 2010). Another study (McKinley et al., 2013) applied tES to a visual search task and found that tES accelerates learning of threat detection skills and improves target acquisition accuracy. However, tES was not able to provide any benefit until users familiarized with the task, making the whole procedure slower than traditional training approaches.

### 3.9. Complex Problem Solving

Problem-solving is another mental ability that can be enhanced by neuroscience technologies. For instance, Cerruti and Schlaug (2009) showed that tDCS could improve performance in the Remote Associates Test, a verbal problem-solving task involving the presentation of three cue words that are linked by a fourth word, which a participant needs to correctly guess. An easy instance of this is “aid,” “rubber,” and “wagon” that are cues for “band,” while a difficult version is “stick,” “maker,” and “point” that are cues for “match.” In Chi and Snyder (2012) tDCS was shown to enable 40% of participants to solve a difficult puzzle requiring connecting nine dots organized in a 3 by 3 square grid with four straight lines, drawn without lifting pen from paper or retracing a line. In the absence of stimulation no participant could solve the problem. Another example of tDCS-based problem-solving augmentation was presented in Dockery et al. (2009), where the speed at which a planning task was performed was improved with no loss in term of accuracy. The task used was the Tower of London test consisting in presenting two boards with pegs and several beads of different colors inserted in the pegs and asking a participant to plan the stacking/unstacking moves required to transform one board configuration into the other.

Naturally also TMS neurostimulation can achieve human performance improvements, although enhancing high-level cognition, including problem solving, is a currently still an objective (Parasuraman and McKinley, 2014; Nelson et al., 2015).

## 4. Ethical Issues

Advances in neuroscience and the development of neuroscience technologies have increasingly raised new and unique ethical issues (“neuroethics”), in addition to the more traditional aspects related to human participation in research studies. This topic is covered in great detail in a recent Royal Society report (Chan and Harris, 2012) and in even more recent dedicated literature (Clark, 2014; McCullagh et al., 2014; Hildt, 2015). For these reasons, here we only mention the most important ethical problems associated with human cognitive augmentation and BCIs referring the reader to such publications for more information.

### 4.1. Mind Reading and Privacy

Some issues are related to the potential of neuroimaging techniques—such as EEG or fMRI—for detecting, mapping and interpreting neural activity of an individual in specific circumstances. Thus, such techniques may raise concerns in relation to free will, privacy, agency, and liability, given their potential ability to “read” or otherwise “assess” someone's thoughts, emotions, states or attitudes, potentially affecting people's moral or social behavior (Chan and Harris, 2012).

In fact, “mind reading” has been often mentioned as a potential risk of BCIs. Of course, at present there is nothing further from the truth: most BCIs can interpret user intentions and commands only if the user wants to make such intentions and commands “heard” to the BCI (e.g., via imaginary movements). This is not very different from what happens with verbal communication, where thoughts are translated into sequences of “instructions” for the larynx only if a person willingly activates speech motor control areas in the brain. However, mind reading is potentially a concern, particularly in relation to privacy violations, when mental activity is monitored, such as in neuroergonomics, passive BCIs or hyperscanning. Mind reading, as such, is not possible as yet, however, given how neurotechnologies are developing and given that the use of invasive neurotechnologies might become more common in the future, it might become an area where clear ethical regulation needs to be developed. This becomes even more problematic in the area of brain-to-brain communication, where the involuntary transfer of thought from one mind to another might become a possibility in the future (Trimper et al., 2014), as might the voluntary control from a decoder to a receiver. For example, the same brain patterns that are modulated by an individual using BCI techniques (as described in sections 3.1.1 and 3.1.2) could be transferred to a receiver thus controlling what they communicate, their use of external devices or prosthetic devices.

### 4.2. Agency, Responsibility, and Liability

Other authors (McCullagh et al., 2014) raised the issue of responsibility (for example, when a new BCI is unsuccessful, was it due to a technology failure or an uncooperative or otherwise unsuitable participant?). Because BCIs are not 100% accurate, there is also of course an issue of liability (if the BCI incorrectly issues a command which causes harm or financial loss, who should be legally responsible for this? The designer of the BCI or the user?).

The advent of brain-to-brain communication devices then amplifies issues associated with agency, responsibility and liability of actions (Trimper et al., 2014; Hildt, 2015). For instance, when an encoder's brain and decoder's brain are connected and the decoder initiates a sequence of actions, who is responsible for them? With the number of possible messages sent to a decoder and their complexity potentially increasing, and possibly involving movement, memory, emotion, it will be more and more complex to understand agency, responsibility and liability.

An additional aspect that can be associated with the use of neuroscience technologies is the potential transfer of moods, memories or personality characteristics from an individual to another (McCullagh et al., 2014).

### 4.3. Safety and Invasiveness of Brain Stimulation

Other issues are related to the possibility of actually changing and affecting brain activity using a variety of brain stimulation techniques to enhance cognitive abilities (such as those discussed in previous sections). In ethically evaluating technologies based on neurostimulation, one needs to consider the uncertainty regarding safety, in particular with invasive methods (e.g., DBS) and ask whether they are safe or safer than other methods currently in use (e.g., non-invasive neurostimulation) (Clark, 2014). For example, when brain stimulation is used to enhance cognition, there is currently little understanding about how safe such stimulation is for use on a regular basis and for prolonged time intervals (Wurzman et al., 2016). In addition, and specifically related to tES and TMS (which are often used in cognitive enhancement), there is the issue of invasiveness. Normally both TMS and tES are considered non-invasive types of stimulation, in that they do not require surgery or direct stimulation of the cortical tissues, and we agree with this classification. However, others feel that tES and TMS are somehow in between between invasive and non-invasive (e.g., Davis and van Koningsbruggen, 2013).

### 4.4. Society

Another concern is related to the benefits of neuroscience technologies for the wider society: are the costs justified by the benefits? Even when these technologies do not present serious risks, it is often unclear whether their use brings benefits to society. Finally, another argument involves the potential risks imposed by an increasing dependence on neuroscience technologies, which might have unforeseen negative societal effects (Rees, 2003).

## 5. Future Prospects

This section will look at future prospects for human cognitive augmentation based on neurotechnologies.

### 5.1. A Roadmap for Human Augmentation Neurotechnologies

Neuroscience for human augmentation is one of the most promising emerging technologies for the future. However, human augmentation is still widely underrepresented in existing roadmaps recently published in the literature (Future Brain/Neural Computer Interaction, BNCI,B; Brunner et al., 2015; Wiseman, 2016). Therefore, we developed a roadmap representing the current state of the art and probable future developments of different neuroscience technologies and human augmentation applications. These predictions are based on three key factors: (a) how each technology/application has developed in the last two decades; (b) the number of publications or research studies using each technology for the different applications; (c) the predictions made in Future Brain/Neural Computer Interaction (BNCI,B), Brunner et al. (2015), and Wiseman (2016).

**Figure 2:**
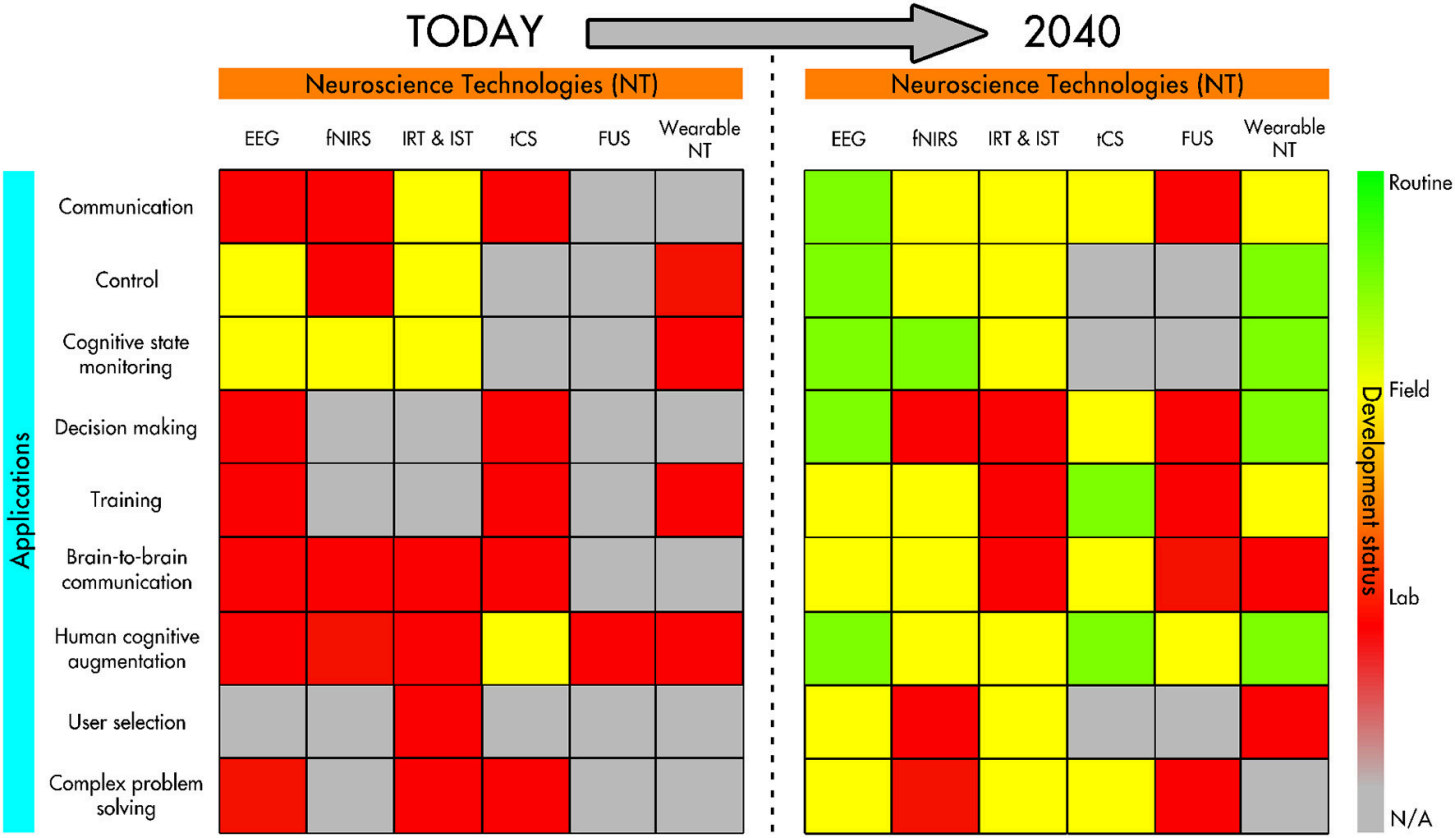
Roadmap of the development of neuroscience technologies for different human augmentation applications. IRT, Invasive Recording Technology; IST, Invasive Stimulation Technology.

The roadmap is shown in Figure 2, where the left grid shows the current state of the art, while the right grid shows our predictions as to the state of the art in 2040. In the figure, “Routine” (green) means that the technology is used in everyday life, meaning that most of the ethical and technological barriers relating to that technology have (or will have) been overcome. “Field” (yellow) indicates technologies tested in the field in preparation for being rolled out for general use, with certain issues (mainly ethical) still to be solved. “Lab” (red) designates applications in which the technology is currently under development/investigation. “Not Applicable (N/A)” (gray) indicates that the technology is not (or will not be) used for a particular application.

In the next three sections we will look in more detail to the future of neuroscience technologies for recording and stimulating brain activity, of human cognitive augmentation applications and of neuroethics.

#### 5.1.1. Future of Neurotechnologies for Recording and Stimulating Brain Activity

The neuroscience technologies shown in the roadmap in Figure 2 are those presented in section 2 plus wearable neuroscience technologies, since they appear to be a natural evolution of current technologies that will likely be available in the future.

If past trends are the best predictors of future ones, then both significant improvements to existing technologies and new technologies for recording and stimulating brain activity should be expected in the medium to long term. It is likely that the development of each technology will continue over the next two decades, considering the advantages provided by each (see section 2). Non-invasive techniques will still remain central thanks to their continuous development and increased reliability. At present and in the context of potential applications, EEG, and fNIRS possibly offer the best compromise, particularly thanks to their portability, low-cost, non-invasiveness, and widespread adoption in current BCI and neuroergonomics studies. In the future, EEG is likely to become even more practical if dry electrode technology continues to develop at its current pace (Lopez-Gordo et al., 2014).

However, it is expected that over time invasive brain-activity observation techniques, such as ECoG or implanted electrodes, will become progressively more ethically and medically acceptable, particularly if the long term risks associated with their presence inside the body are proven minor. After all, many forms of body modification are already accepted both for medical (e.g., pace makers, laser vision-correction, and cochlear implants) and aesthetic (e.g., face-lifts, body piercing, or tattoos) reasons. If that is the case, invasive techniques will offer a more precise and effective way of observing brains in action, particularly if the recent trends in recording technology (Qiao et al., 2016; Pesaran et al., 2018) continue.

In relation to neurostimulation technologies, at present and in the context of potential applications, the best compromise is offered by tES, which is portable, generally cheap, and non-invasive. For brief exposures, this technology appears to be low-risk, and the recent development of a higher-definition form of tES suggests that further improvements are forthcoming. Energy considerations make it difficult to imagine how TMS could ever become portable. In the future, it appears as if FUS may become superior to both technologies in terms of resolution and portability (portable ultrasound devices already exist on the market, suggesting the feasibility of making FUS portable), but it is unclear whether it will ever be possible to stimulate multiple sites and large areas of the brain at once. If invasive techniques, such as implanted electrodes, ever become acceptable, they will of course offer a more direct and precise way to modulate brain activity.

#### 5.1.2. The Future of Human Augmentation

The roadmap in Figure 2 shows the trend of development of the main applications of neuroscience technologies reviewed in section 3. In the forecast horizon of the roadmap (a period of over two decades), an acceleration of these developments is likely, particularly as ethical, medical, and technological obstacles are progressively removed, paving the way to making invasive brain-activity observation technologies viable. In general, it can be expected that BCIs for communication and control will have improved sufficiently to become routinely used particularly in domains where higher than musculoskeletal reaction times are important or where covert communication is required. However, it is also clear that within this time frame many neuroscience technologies for augmenting human performance will continue to transition (having currently just started) outside the lab for field testing with some even in routine use. For instance, significant progress can be expected to be made in innovative applications in training and selection of personnel, decision-making, cognitive monitoring, and situation awareness, given their current initial successes.

Finally, it should be noted that all forms of enhancement based on neurostimulation look, at present, extremely promising, although they still present risks. For instance, facilitation of one function might be expected to be associated with loss of some other, often unknown function. Furthermore, research on the long term effects of such technologies is lacking. For these two reasons, the future of stimulation technologies is harder to predict as their currently formidable expansion would likely come to a sudden halt if future research reveals that they have severe permanent side effects.

#### 5.1.3. Ethics

Fear of change and of the unknown is understandable. Fuelled by this, often the ethical debate appears to focus on what is conceivable, rather than on what is scientifically foreseeable (i.e., there being only technological limits to its attainment) and what is already reality. This may lead to illogical and unexpected outcomes. As it is difficult to predict the exact future trajectory of neuroscience, neuroergonomics, BCIs, and human augmentation technologies, it is also difficult to predict how neuroethics, i.e., how society, will look at such technologies. It, therefore, critically important to track ethical implications, particularly in areas such as mind reading and privacy, agency, responsibility, and liability. Given the recent trajectory of neuroscience, BCIs, neuroergonomics, brain-to-brain-communication and neural engineering, and their formidable expansion, such applications may one day become reality, and, so, they deserve to be ethically debated.

However, none of the ethical issues mentioned in section 4 appear to be a show stopper for human enhancing neurotechnologies. Some issues can be tackled technologically. For instance, preventing (future) BCIs from inadvertently communicating private thoughts or emotions could easily be achieved by requiring users to issue a particular sequence of mental commands (akin to the password required to unlock the screen of a smartphone) to switch the BCI on and off (in fact this is already an element of the family of so called “self-paced” BCIs). For other issues, it is possible to simply apply ethical standards already accepted in similar situations (for example Smidt, 2000). One can expect that over time ethical thinking will progressively change as a result of society being exposed to neuroscience technologies for human augmentation resulting in further acceleration in their development and adoption. Nonetheless, as neurotechnologies evolve, the development and adaptation of clear ethical regulation is becoming more and more pressing.

## Author Contributions

All authors listed have made a substantial, direct and intellectual contribution to the work, and approved it for publication.

### Conflict of Interest Statement

The authors declare that the research was conducted in the absence of any commercial or financial relationships that could be construed as a potential conflict of interest.

## Footnotes

^1^ITR measures how many bits of information are transferred across a communication channel per unit of time.
